# Heidelberg Neuro-Music Therapy Restores Attention-Related Activity in the Angular Gyrus in Chronic Tinnitus Patients

**DOI:** 10.3389/fnins.2017.00418

**Published:** 2017-07-20

**Authors:** Christoph M. Krick, Heike Argstatter, Miriam Grapp, Peter K. Plinkert, Wolfgang Reith

**Affiliations:** ^1^Department for Neuroradiology, Saarland University Hospital Homburg, Germany; ^2^German Research Centre for Music Therapy Research Heidelberg, Germany; ^3^Department of Otorhinolaryngology, Head and Neck Surgery, University Hospital for Ear, Nose, and Throat, University of Heidelberg Heidelberg, Germany

**Keywords:** tinnitus, fMRI neuroimaging, Heidelberg model of music therapy, tinnitus distress, tinnitus treatment, attention

## Abstract

**Background:** Tinnitus is the perception of a phantom sound without external acoustic stimulation. Recent tinnitus research suggests a relationship between attention processes and tinnitus-related distress. It has been found that too much focus on tinnitus comes at the expense of the visual domain. The angular gyrus (AG) seems to play a crucial role in switching attention to the most salient stimulus. This study aims to evaluate the involvement of the AG during visual attention tasks in tinnitus sufferers treated with Heidelberg Neuro-Music Therapy (HNMT), an intervention that has been shown to reduce tinnitus-related distress.

**Methods:** Thirty-three patients with chronic tinnitus, 45 patients with recent-onset tinnitus, and 35 healthy controls were tested. A fraction of these (21/21/22) were treated with the “compact” version of the HNMT lasting 1 week with intense treatments, while non-treated participants were included as passive controls. Visual attention was evaluated during functional Magnet-Resonance Imaging (fMRI) by a visual Continous Performance Task (CPT) using letter-based alarm cues (“O” and “X”) appearing in a sequence of neutral letters, “A” through “H.” Participants were instructed to respond via button press only if the letter “O” was followed by the letter “X” (GO condition), but not to respond if a neutral letter appeared instead (NOGO condition). All participants underwent two fMRI sessions, before and after a 1-week study period.

**Results:** The CPT results revealed a relationship between error rates and tinnitus duration at baseline whereby the occurrence of erroneous “GO omissions” and the reaction time increased with tinnitus duration. Patients with chronic tinnitus who were treated with HNMT had decreasing error rates (fewer GO omissions) compared to treated recent-onset patients. fMRI analyses confirmed greater activation of the AG during CPT in chronic patients after HNMT treatment compared to treated recent-onset patients.

**Conclusions:** Our findings suggest that HNMT treatment helps shift the attention from the auditory phantom percept toward visual cues in chronic tinnitus patients and that this shift in attention may involve the AG.

## Introduction

Tinnitus is the perception of a phantom sound, such as a pure tone, ringing, buzzing, or other noise, without external acoustic stimulation. However, while the occurrence of tinnitus may be often disturbing, it does not necessarily cause the disease pattern of a mental suffering (Malouff et al., 2011). Conversely, the duration since the tinnitus onset and the gender seem influence the individually reported mental distress (Seydel et al., 2013). Thus, the etiology of tinnitus-related distress depends on a number of poorly understood factors (Møller, 2016) that has led to several models and theories on the pathophysiology of tinnitus (Henry et al., 2014). Findings from Positron Emission Tomography (PET) measurements showed an involvement of auditory and non-auditory networks for maintaining the tinnitus percept (Mirz et al., 1999). The awareness of tinnitus activates those brain centers that are also involved in conscious acoustic perception (Dehaene and Changeux, 2011). Presumably prefrontal and parietal attention networks play a crucial role in conscious perception. The inferior parietal lobe has been found to be a key region for the awareness of an acoustic percept (Sadaghiani et al., 2009). Recent tinnitus research suggested a relationship between these attention processes and maintenance of tinnitus (Roberts et al., 2013). Husain et al. (2015) observed that tinnitus patients' attention networks showed more activation during visual than auditory tasks. These findings suggested a need for rising engagement of the attention system in tinnitus patients while focusing on visual stimuli. It is unclear, however, whether these perceptual changes arose immediately with the tinnitus onset or whether they were gradually provoked by the persisting tinnitus percept. Recent findings on tinnitus distress suggested that the distress-related higher mental effort for attention control may arise primarily from focusing on self-referred emotional signals that were triggered by a network consisting of the amygdala, the anterior cingulate cortex, the insula, and the parahippocampal area (Vanneste et al., 2010). Patients' attention thus shifts from the outside world toward the inner “mental pain” caused by the tinnitus distress (Malouff et al., 2011). Mohamad et al. (2016) summarized attention-related studies in tinnitus sufferers: That review did not tend to generally impaired attention due to tinnitus but some evidence that tinnitus patients performed poorer on selective attention in visual tasks compared to normal hearing controls (Hallam et al., 2004; Stevens et al., 2007), but non-significant findings were mentioned, too (Heeren et al., 2014). Hence, the observations so far remained unclear whether and how the tinnitus percept may claim resources for the visual attention. Selective attention in the visual domain and the respective brain responses can be measured by a Continuous Performance Task (CPT) which requires sustained attention to a sequence of visual stimuli (Hester et al., 2004). In this context, reaction time (RT) and error rates for responses can be measured to assess effects of a musical intervention (Guo et al., 2015).

The inferior parietal lobe plays a key role in conscious awareness of acoustic and visual cues (van Gaal et al., 2011; Igelström et al., 2016). Humphreys and Chechlacz (2015) described a network for attentive visual search which includes the angular gyrus (AG), the middle occipital gyrus, superior and middle temporal gyri, and the insula. The latter is also known as part of the tinnitus network, whereas the temporal regions and the AG are involved in self-awareness and self-perception (De Ridder et al., 2014). Hester et al. (2004) found these regions to be systematically involved in self-monitoring during visual CPT. The AG appears to play a crucial role for binding the attention to the individually most salient sensory stream (Taylor et al., 2011). Visual attention can be disrupted by suppressing AG activity using Transcranial Magnet Stimulation (TMS; Taylor and Thut, 2012). If the AG is involved in driving awareness toward the most salient sensory modality, the attention focus may be strongly driven toward the auditory system in case of tinnitus (De Ridder et al., 2014; Husain et al., 2015). Resting-state functional MRI scans revealed higher amplitude of low-frequency fluctuations in the AG and other attention-related areas (Chen et al., 2015). PET measurements confirmed these findings showing higher AG activity in chronic tinnitus patients (Song et al., 2012). Binding the attention to the auditory sensation in turn may explain the attention deficits in visual tasks as well as the attention conflict in the auditory domain in tinnitus patients (Araneda et al., 2015; Li et al., 2016). Using PET measurements, Plewnia et al. (2007) observed both tinnitus-related hyperactivity in the right AG and TMS-related attenuation of tinnitus after stimulation of this area. This finding indicated that the AG is a key region in understanding the erroneous focus on the phantom noise in clinically decompensated tinnitus patients.

A number of therapy options have been developed for tinnitus treatment, comprising Tinnitus Retraining Therapy, Cognitive Behavioral Therapy, Progressive Tinnitus Management, Biofeedback, Education, and Relaxation Therapies (Herraiz et al., 2007; Hesse and Laubert, 2010; Hesser et al., 2011; Folmer et al., 2014; Grewal et al., 2014; Myers et al., 2014). Most therapies aim to dissuade patients auditory self-monitoring. In those studies, however, there was little consistency in the therapeutic effects and their scientific verifiability. The statistically weak or inconsistent observations may reflect variation in the aberrant mental states of tinnitus patients. With respect to long-lasting neuroplastic changes, starting with the onset of tinnitus (Mühlau et al., 2006; Landgrebe et al., 2009; Schneider et al., 2009; Husain et al., 2011; Leaver et al., 2012; Boyen et al., 2013; Schecklmann et al., 2013), one can assume that the duration of tinnitus is one of several factors for the variable effects in tinnitus relief (Malouff et al., 2011). Relief is not directly related to the loudness of the phantom sound, but to the level of “catastrophizing” its affective experience (Møller, 2016). This in turn corresponds to the strength of an emotionally driven attention shift in tinnitus' pathophysiology causing tinnitus-related distress (Vanneste et al., 2010; Ueyama et al., 2013). The compact approach of the Heidelberg Neuro-Music Therapy (HNMT) has been shown to reduce tinnitus-related distress as assessed by a controlled trial (Argstatter et al., 2015). Its effects have also been shown to be maintained over a long time (Argstatter et al., 2012).

In the current study, we implemented a task that has previously been used in our research unit for examination of adults with attention deficit syndrome, the visual CPT (Schneider et al., 2010). The CPT was implemented as GO/NOGO task, allowing the observation of attention and inhibition control. Attention difficulties can be detected by omitted GO stimuli, whereas decreased inhibition control results in erroneous NOGO reactions. The current paper aims to show the effects of tinnitus duration on the control of visual attention and the effects of HNMT on the visual attention network in tinnitus patients. Thus, we assessed the attention binding to the visual cue that required the participant's decision for action or inhibition. We postulated that HNMT will improve the visual attention in those patients with initial attention impairments.

## Materials and methods

### Participants

Both recent-onset patients unsuccessfully completing the standard clinical treatment (tinnitus persisting for a maximum of 6 months) and patients with chronic tinnitus (duration of at least 6 months) were invited for participation. Thirty-three patients with chronic tinnitus (12 females), 45 patients with recent-onset tinnitus (19 females), and 35 healthy controls (18 females) participated in the trial (Table 1). The group of healthy controls was enrolled after recruitment of the tinnitus groups, according to the patients' gender ratio and age distribution. Mean age was 47.6 ± *SD* 10.4 years in the chronic tinnitus group, 43.1 ± *SD* 10.5 years in the recent-onset tinnitus patients, and 43.4 ± *SD* 14.5 years in healthy controls (Table 1). In the group of recent-onset patient there was less variance with respect to the tinnitus duration (mean 8.1 weeks ± *SD* 1.6 weeks), since those participants were systematically included immediately after the standard treatment by otorhinolaryngology. In contrast to this, tinnitus duration in the chronic cases ranged from 1 to 14 years (mean 5.26 years ± *SD* 4.1 years). All participants underwent clinical examination and audiometric testing. The tinnitus patients were additionally examined by a pre-participation evaluation based on the TRI-recommendations (audiological testing, otolaryngological examination, and psychological intake interview). Patients were excluded if the tinnitus was related to anatomic lesions of the ear, retro-cochlear lesions, or to cochlear implants. Further exclusion criteria included clinical diagnosis of a comorbid severe mental disorder, clinical diagnosis of Menière's Disease, severe hyperacusis, or severe hearing impairment (>40 dB) in the tinnitus frequencies region. The latter criterion was defined to allow the participation of HNMT without hearing aids.

**Table 1:** Participants (*n* = 113) and therapy participation.

		**Tinnitus**			**Sum**
		**Controls**	**Recent-onset**	**Chronic**	
Music therapy and tinnitus profile	None (*n*)	13 (8f/5m)	24 (10f/14m)		37
	Frequency (*SD*)	None	6,376 (3,176)		
	Duration (*SD*)		0.16 y (0.03)		
	Standard (*n*)			12 (3f/9m)	12
	Frequency *(SD)*			6,200 (3,615)	
	Duration *(SD)*			6.09 y (4.05)	
	Compact (*n*)	**22 (10f/12m)**	**21 (9f/12m)**	**21 (9f/12m)**	**64**
	Frequency *(SD)*	None	5,102 (2,332)	6,785 (2,547)	
	Duration *(SD*)		0.16 y (0.04)	4.80 y (3.56)	
Sum		35	45	33	113

*The HNMT was offered weekly (standard) or condensed in 1 week (compact). For fMRI comparison of HNMT effect only participants undergoing the compact treatment were included (n = 64; bold). Behavior observations, however, comprised reaction data of all participants at first measurement (before the study period)*.

The study was conducted in accordance with the Declaration of Helsinki and approved by the local Saarland (Germany) ethics committee (ID-number 111/11). A complete clinical study protocol was compiled according to the ICH guidelines for good clinical practice.

### Tinnitus questionnaire (TQ)

Psychological complaints were assessed using the German version of the “Tinnitus Questionnaire” (TQ) as described by Goebel and Hiller (1998). This well-validated inventory comprises 52 items and records tinnitus related complaints. The items can be aggregated to variables representing the dimensions of mental distress: emotional and cognitive load, tinnitus duration, hearing impairments, sleep disturbance, and somatoform disorders. The global TQ-score ranges between the minimum score of 0 and the maximum score of 84, in which high values indicate high tinnitus related distress. Four levels of severity include: mild (0–30), middle (31–46), severe (47–59), and very severe (60–84) affliction. Cross-correlation between TQ and Tinnitus Handicap Inventory (THI) was reported by Zeman et al. (2012) yielding a Pearson's correlation coefficient of 0.87.

The patients' distress was examined by TQ at the time point of the pre-participation evaluation (T0) as well as before (T1) and after (T2) the study period. In the tinnitus groups, we included adult patients who were diagnosed with disturbing tinnitus (TQ-score > 30), but not completely decompensated tinnitus (TQ-score < 64). At T0, the tinnitus distress as measured by TQ was 43.2 ± 9.6 *SD* in chronic patients and 37.3 ± 15.8 *SD* in recent-onset patients. Since the tinnitus duration is regarded in the TQ score, there was a systematic difference between recent-onset and chronic tinnitus.

### Intervention (HNMT)

All participants from the three groups, chronic and recent-onset tinnitus patients as well as healthy controls, were included in the examination of visual attention, but the groups were randomly divided in different treatment cohorts, comprising the participation in the “compact” HNMT in each group. The other option consisted in either no treatment or weekly sessions (“standard” protocol) instead of the compact treatment over 5 days. So, in each group of participants, treatment cohorts with comparable sample sizes were selected for undergoing the compact HNMT application. Thus, 21 chronic tinnitus patients (9 females, tinnitus duration 250 weeks ± 187 *SD*), 21 recent-onset tinnitus patients (9 females, tinnitus duration 8.14 weeks ± 1.85 *SD*), and 22 healthy controls (10 females, no tinnitus) underwent the compact HNMT.

For examination of interaction between tinnitus duration and visual performance, the initial behavioral measurements at T1 from all participants, comprising both treated and non-treated subgroups, were regarded. At this time the conditions were comparable for all participants, since there wasn't yet an influence from the subsequent therapy option. Hence, evaluations from this time point were independent from the therapy option and, hence, used to generally assess influences from the individually pre-existing or not existing tinnitus to the visual attention.

**Figure 1:**
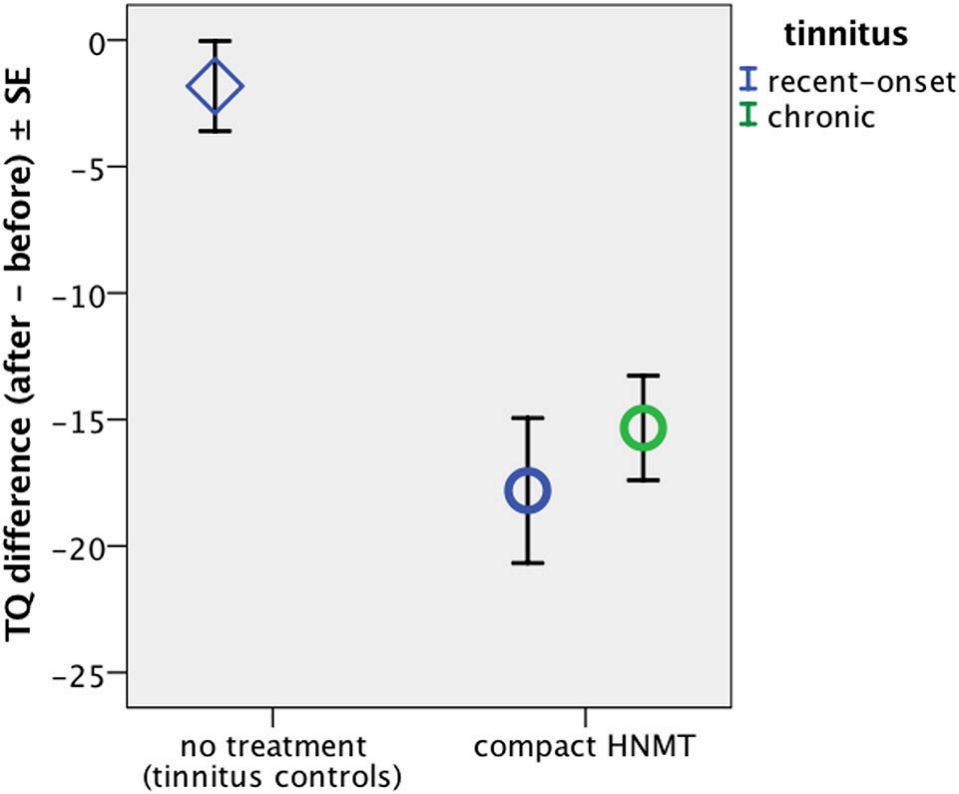
Change in subjective tinnitus distress as measured by TQ. Krick et al. (2015) found that HNMT caused a major improvement in tinnitus distress in recent-onset tinnitus patients (blue circle), as measured by a reduction of near 18 on the TQ scale, an improvement which was not observed in non-treated passive control patients (rhomb). Here we measured the HNMT effect in chronic tinnitus sufferers (green circle), revealing a similar decrease on the TQ score by 15.3 ± *SD* 9.5 (*n* = 21; *Z* = −4.3; *p* < 0.0001). There was no significant difference in the therapy-induced TQ reduction between treated chronic and treated recent-onset patients (*n* = 21/21; *Z* = −0.4; *p* > 0.6).

The subgroups treated with the compact HNMT (Table 1, marked in bold text) were compared to reveal the duration-specific effect among therapy-related effects on the attention network. The sub-groups of recent-onset tinnitus patients and the “treated” healthy controls have been already included in a previously reported study on structural effects from HNMT, in which we found that HNMT caused a major improvement of 17.7 points on the TQ scale in treated tinnitus patients (Figure 1, Krick et al., 2015). In the current study, we analogously evaluated the effects of HNMT in chronic tinnitus patients. As the HNMT has been found to rapidly reduce tinnitus distress (Grapp et al., 2013; Krick et al., 2015), this therapy can be used to assess distress-related effects on brain activation during a short study period.

The study protocol of the compact version of HNMT consisted of 9 consecutive 50-min sessions of individualized therapy, comprising acoustic training for frequency discrimination, auditory attention control tasks, and guided exercises for mindfulness and distress regulation. Therapy took place on 5 consecutive days (Monday to Friday) with two therapy sessions per day. Music therapy can be divided into two main categories: receptive (music listening based) and active (music making). Each morning and each afternoon session lasted for 50 min, in which there were 25 min of active music therapy and 25 min of receptive music therapy. Two trained music therapists carried out the therapy, in which one therapist performed the active modules and the other performed the receptive modules. The interventions were structured into treatment modules of directive counseling, habituation training, and stress management. A more detailed description can be found in Argstatter et al. (2015) and Grapp et al. (2013).

### Experimental setup

Independently from the assignment to the treatment cohorts, all participants performed a letter-based CPT twice, during two fMRI sessions at T1 and at T2. Capital letters “A” through “H” were used as “neutral” stimuli in a quasi-random order. Letters “O” and “X” were used as alerting events inserted in the letter stream. Each letter was presented for 420 ms on a screen. Participants were instructed to watch the rapid sequence of letters and to look out for the letter “O” as an alarm stimulus. If the next letter following the “O” was an “X,” they were asked to press a button (GO condition). In all other cases, the participants were instructed not to press the button. Especially if the letter “O” was followed by a letter different from “X,” no reaction (NOGO condition) was required. The response button was counterbalanced for left and right hand in all groups.

Each condition consisted of 30 trials of GO or NOGO events which were randomly inserted among the presentation of 1800 letters over 12.6 min. The letters were projected through a window of the Magnet-Resonance (MR) cabin on a screen placed behind the head-side opening of the scanner. A mirror on the top of the head coil allowed viewing the letters in a light square on black background. The presentation was controlled by a computer synchronized with the imaging pulses.

### Data acquisition and data analysis

The 3 tesla MR scanner “Skyra” (Siemens) was used for imaging. Functional scan parameters of the Blood Oxygen Dependent (BOLD) imaging were set to time-to-repeat (TR) of 2.2 s, echo time (TE) to 30 ms, and flip angle of 90°. Thirty slices of 3 mm thickness with a 0.75 mm gap to the next slice covered the whole brain. Each slice was scanned into a 94 × 94 matrix resulting in a voxel volume of 2 × 2 × 3 mm^3^. For each run, 348 scans were acquired, including 4 prescans to compensate for magnetization saturation effects. The prescans were later discarded from data processing.

The functional BOLD imaging was conducted at T1 and T2. Two high-resolution anatomical scans over the whole brain were also measured at T1 and at T2, using a Magnetization Prepared Rapid Acquisition of Gradient Echoes (MPRAGE; Mugler and Brookeman, 1990) sequence. This 3D brain image resulted in isometric voxel dimensions of 0.9 × 0.9 × 0.9 mm^3^.

Thus, functional and anatomical MRI (fMRI) scans were each conducted in two consecutive MRI sessions before and after the 1-week study period, meaning that all participants underwent the GO/NOGO CPT twice, at T1 and T2. The same hand and the same procedure were used in both consecutive measurements. During the MRI sessions, double ear protection (earmuffs and head phones) was applied to reduce scanner noise. For compensating ametropia, MRI-compatible glasses with adapted diopter for each eye were offered if necessary.

Preprocessing and modeling of MRI measurements were executed using standard procedures of *Statistical Parametric Mapping* (SPM8, Wellcome Trust Centre for Neuroimaging, London). Preparation of the functional scans included:

- slice time and motion correction of functional scans;
- segmentation of the anatomical scan;
- co-registration of the resulting gray matter (GM) compartment to the mean image of corrected functional scans;
- co-registration of the anatomical image to the position of the functional mean image;
- determination of normalization parameters using the anatomical image;
- application of normalization parameters to the functional scans with fitting to the template of the Montreal Neurological Institute (MNI space);
- and Gaussian smoothing using an 8-mm radius in each direction.

For general activation regarding to the alarm stimulus, the visual cue “O” from both GO and NOGO trials of the first run were pooled by a conjunction analysis. The effect of HNMT with respect to the tinnitus duration was estimated using both MRI sessions by a flexible factorial model (2 × 2 ANOVA) with “Time” (T1, T2) as within-subject factors and “Group” (chronic, recent-onset, control) as between-subject factors to explore the effect of treatment in the various participant groups. An assumption of interaction between “Time” and “Group” was predicted due to the effect of HNMT and tinnitus duration on the subjects' brain activity.

The GO and the NOGO conditions were modeled using the canonical hemodynamic response function and its first derivative as evoked by the visual alarm stimulus “O.” This first-level “O”-responses of both measurement times (T1 and T2) per participant was included in the flexible factorial model.

Behavioral accuracy and RT from button responses were evaluated separately using SPSS Statistics (IBM). As the error rates from both GO and NOGO conditions did not correspond to a Gaussian distribution, non-parametric tests (Spearman Rho correlation and Wilcoxon test for paired data) were used to investigate the influence from tinnitus duration on the subjects' attention.

## Results

### Therapy effect on subjective distress score (TQ)

Participation in HNMT yielded a reduction of 15.3 ± *SD* 9.5 scale points on the TQ score in chronic tinnitus sufferers (*n* = 21; Mann–Whitney-*U* test: Z = −4.3; *p* < 0.0001) compared to non-treated tinnitus patients (*n* = 22) which showed only a small test-retest effect of −1.8 score points. In chronic tinnitus patients, the subjective tinnitus distress was similarly reduced after HNMT as previously reported for treated recent-onset patients (Krick et al., 2015; Figure 1). When comparing the findings from recent-onset patients with the TQ reduction in treated chronic patients, the effects did not significantly differ between both patient groups (*n* = 21/21; Z = −0.4; *p* > 0.6).

### Visual attention task and behavioral accuracy

**Figure 2:**
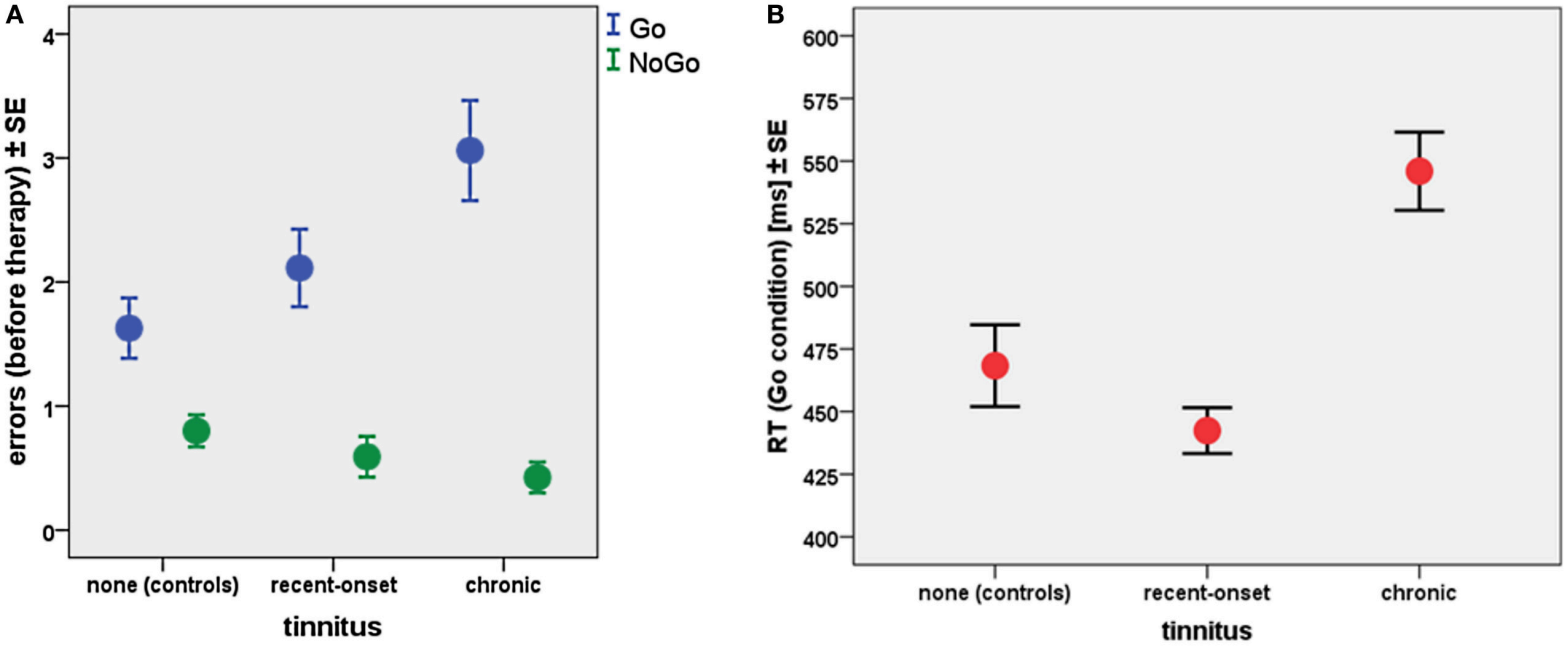
**(A)** Error rates and **(B)** reaction times (RT) related to tinnitus type at T1 for all participants (35 healthy controls, 45 recent-onset tinnitus patients, and 33 chronic tinnitus patients).

Regarding the individual tinnitus anamnesis, there was a significant interaction between tinnitus duration and error rates from GO/NOGO task. At T1, error rates for both the GO condition (Spearman-Rho: +0.27; *p* < 0.005) and the NOGO condition (Spearman-Rho: −0.23; *p* < 0.05) depended on the type of tinnitus: chronic or recent-onset. Error rates from GO trials increased with tinnitus duration, whereas error rates from NOGO trials were diminished with chronic tinnitus (Figure 2A).

Latencies of the observed reactions also yielded effects of tinnitus duration (Figure 2B). Patients with chronic tinnitus exhibited longer RTs compared to healthy controls (Mann–Whitney-U; Z = −5.9; *p* < 0.0001), whereas there was no significant RT difference between controls and patients with recent-onset tinnitus (Mann–Whitney-U; Z = −0.82; *p* > 0.4). A linear regression model showed that the RT latency was delayed by about 42 ms with each year of tinnitus duration (*T* = 7.3; *p* < 0.0001).

**Figure 3:**
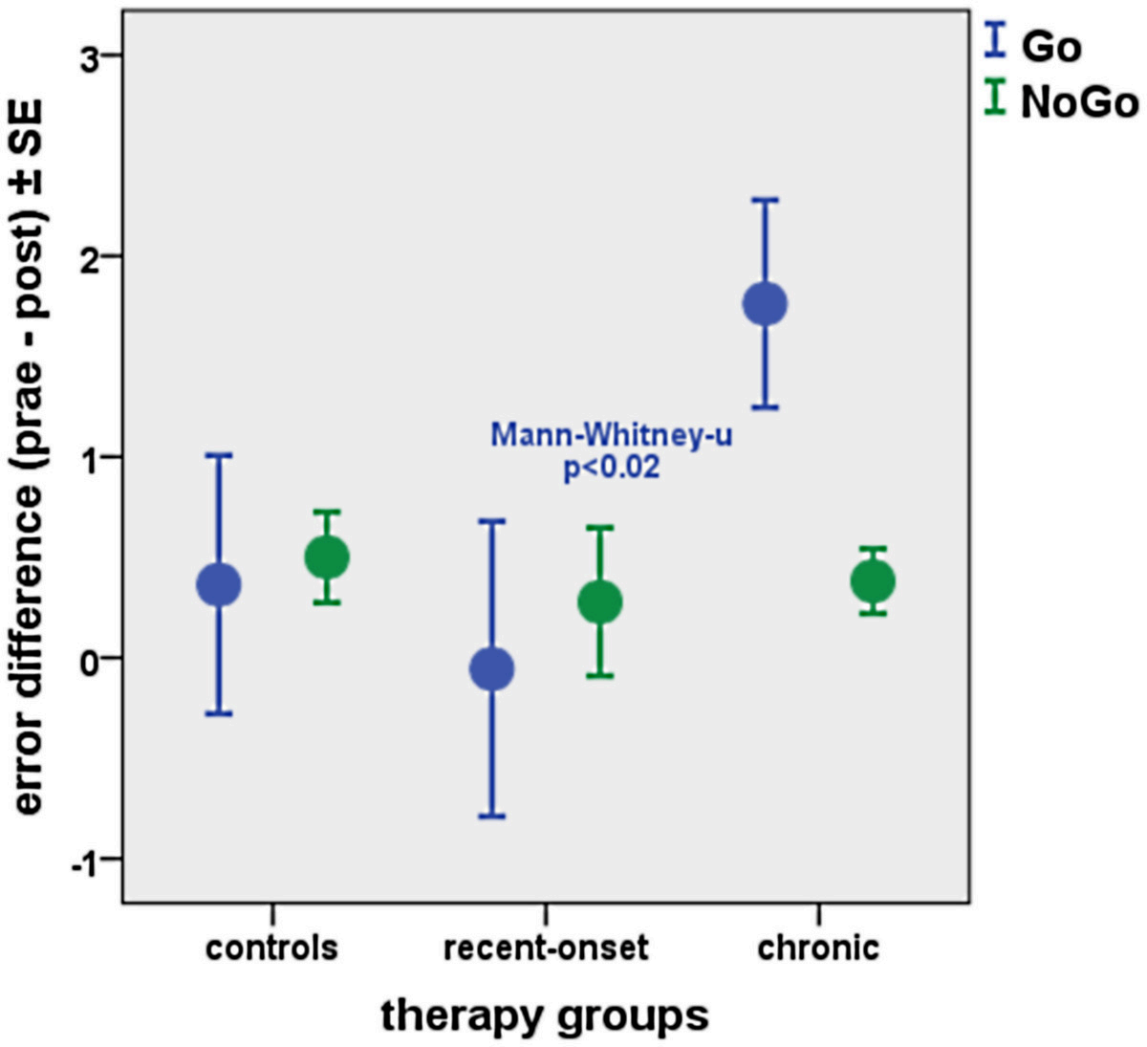
Difference of error rates for GO and NOGO trials before and after HNMT in treated subsamples of healthy controls (*n* = 22), recent-onset tinnitus patients (*n* = 21), and chronic tinnitus sufferers (*n* = 21).

Non-parametric paired Wilcoxon tests were performed to assess differences between HNMT treated and untreated participants. In the non-treated patients, error rates did not differ between start and end of the study period, neither in the GO condition (Wilcoxon Z: −0.01; *p* > 0.99) nor in the NOGO condition (Wilcoxon Z: −1.4; *p* > 0.15). For both groups participating in HNMT, “treated” controls and treated tinnitus patients, there were a measurable effect by the HNMT application in each case, showing each diminishing error rates between T1 and T2. This effect was similar between “treated” controls (GO: Wilcoxon *Z*: −1.68; *p* < 0.1; NOGO: Wilcoxon *Z*: −2.00; *p* < 0.05) and treated patients (GO: Wilcoxon *Z*: −1.83; *p* < 0.1); NOGO: Wilcoxon *Z*: −1.74; *p* < 0.1). However, comparing the subgroups of treated tinnitus patients, the reduction of GO errors was significantly more pronounced in chronic compared to recent-onset tinnitus patients (Mann–Whitney *Z*: −2.3; *p* < 0.02) as shown in Figure 3. In contrast to this, there was no effect on the attention-related errors in non-treated participants (GO: Wilcoxon *Z*: −0.01; *p* > 0.9; NOGO: Wilcoxon *Z*: −1.41; *p* > 0.15).

### Brain activation

**Figure 4:**
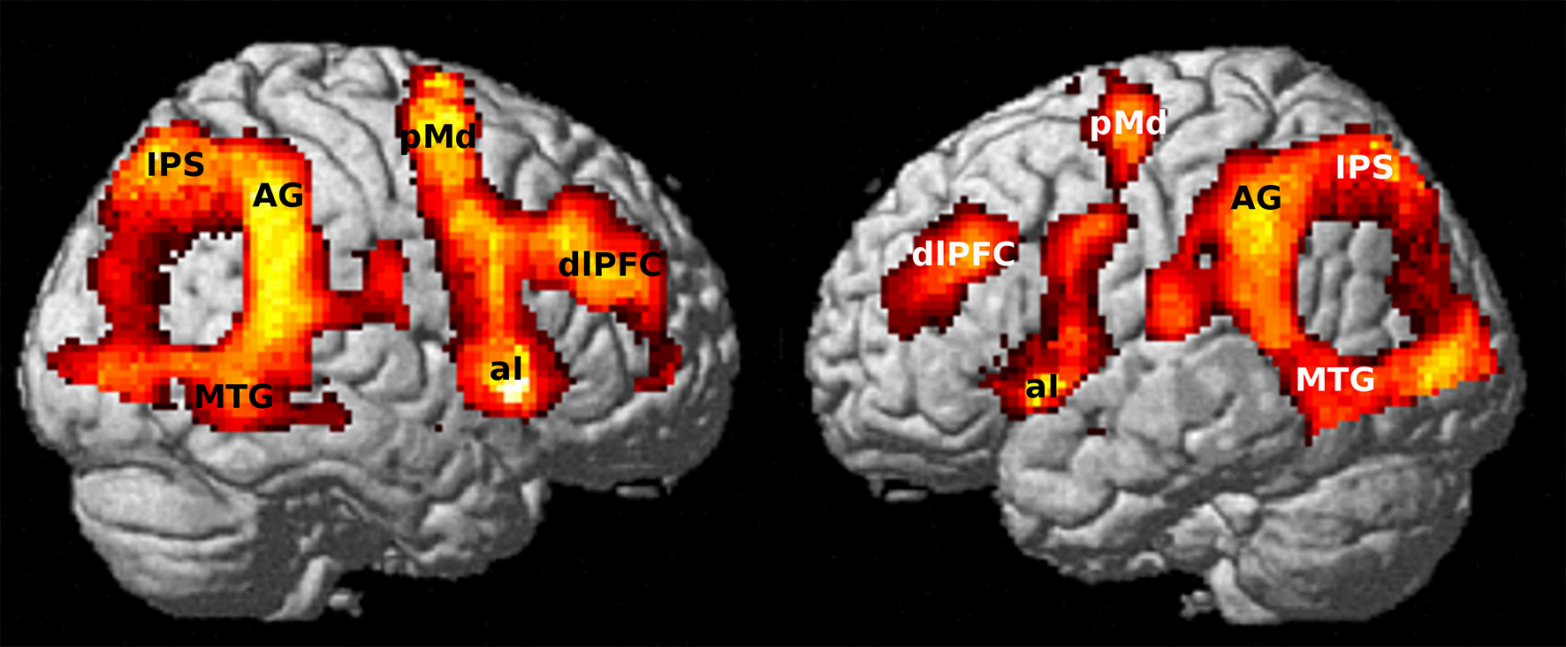
Activated network for to the alarm stimulus (“O”) from the first fMRI session (T1) by conjunction of conditions GO & NOGO (*n* = 113). Maximum activation peaked in the right AG (*p* < 10^−10^ FWE corrected; 20 voxels extent threshold).

BOLD contrast by the effect of visual attention on the cue “O” was derived from the first MRI session (T1) for all participants (*n* = 113). This analysis was realized by a conjunction (comparable with the statistical intersection by an AND operation) of both GO and NOGO conditions, since both conditions were based on the visual cue “O.” We aimed to observe here the brain network for conscious processing and attention binding (Dehaene and Changeux, 2011). Thus, the common activation by the visual cue for both GO and NOGO trials was relevant for observing its interdependency with tinnitus. The brain activations covered dorsolateral inferior frontal areas, the insula, superior and middle temporal regions, the premotor cortex, inferior parietal lobes, and inferior occipital areas in both hemispheres (Figure 4). In this network, activation in the right (MNI: +60/−42/+18) and left (MNI: −26/−62/+46) AG scored highest (Table 2).

**Table 2:** Activated Clusters for the visual cue “O”: (A) general brain activations for the cue “O” and (B) HNMT-induced alterations in relation to tinnitus duration.

**Localization**	**MNI (x y z)**			***p* cluster**
**(A) ACTIVATION DUE TO “O” CUE; CONJUNCTION GO & NOGO, *N* = 113 WHOLE BRAIN ANALYSIS; *P* < 0.00001 FWE CORRECTED**				
R angular Gyrus (AG)	60	−42	18	*p* < 0.001 corr
L angular Gyrus (AG)	−26	−62	46	*p* < 0.001 corr
R intraparietal sulcus (IPS)	34	−62	48	*p* < 0.001 corr
L intraparietal sulcus (IPS)	−32	−50	38	*p* < 0.001 corr
R insula	34	20	4	*p* < 0.001 corr
L insula	−32	18	4	*p* < 0.001 corr
R inferior frontal gyrus (IFG)	46	8	22	*p* < 0.001 corr
L inferior frontal gyrus (IFG)	−32	36	10	*p* < 0.001 corr
R premotor cortex (pMd)	46	8	32	*p* < 0.001 corr
L premotor cortex (pMd)	−28	−12	68	*p* < 0.001 corr
R middle frontal gyrus (MFG)	40	34	32	*p* < 0.001 corr
L middle frontal gyrus (MFG)	−44	32	32	*p* < 0.001 corr
posterior medial frontal gyrus	4	8	52	*p* < 0.001 corr
R middle temporal gyrus (MTG)	50	−52	−6	*p* < 0.001 corr
L middle temporal gyrus (MTG)	−50	−66	−2	*p* < 0.001 corr
**(B) “O” CUE; T2 > T1; CHRONIC (21) > RECENT-ONSET PATIENTS (21) MASKED WITH ROI COVERING THE AG; SMALL-VOL. CORR.; *P* < 0.001 UNCORR**.				
R AG	50	−64	28	*p* < 0.05 corr
L AG	−50	−68	24	n.s.
**Localization**	**MNI (x y z)**			**Cluster size**
**(C) “O” CUE; T2 > T1; CHRONIC (21) > RECENT-ONSET PATIENTS (21) WHOLE-BRAIN ANALYSIS; *P* < 0.001 UNCORR**.				
R angular gyrus (AG)	50	−64	28	236 voxels
L angular gyrus (AG)	−50	−68	24	41 voxels
L middle temporal gyrus (MTG)	−50	−18	−14	55 voxels
R precuneus	10	−56	20	25 voxels
**(D) “O” CUE; T2 > T1; CHRONIC (21) > CONTROLS (22) WHOLE-BRAIN ANALYSIS; *P* < 0.001 UNCORR**.				
L frontal eye field (FEF)	−16	−16	64	79 voxels
R frontal eye field (FEF)	−22	−8	68	191 voxels
R premotor cortex (pMC)	10	26	72	81 voxels
**(E) “O” CUE; T2 > T1; RECENT-ONSET (21) > CONTROLS (22) WHOLE-BRAIN ANALYSIS; *P* < 0.001 UNCORR**.				
no suprathreshold clusters				

*The cluster-related significance (p cluster) was calculated after Family-wise Error (FEW) correction for multiple comparisons (abbr. “corr”)*.

**Figure 5:**
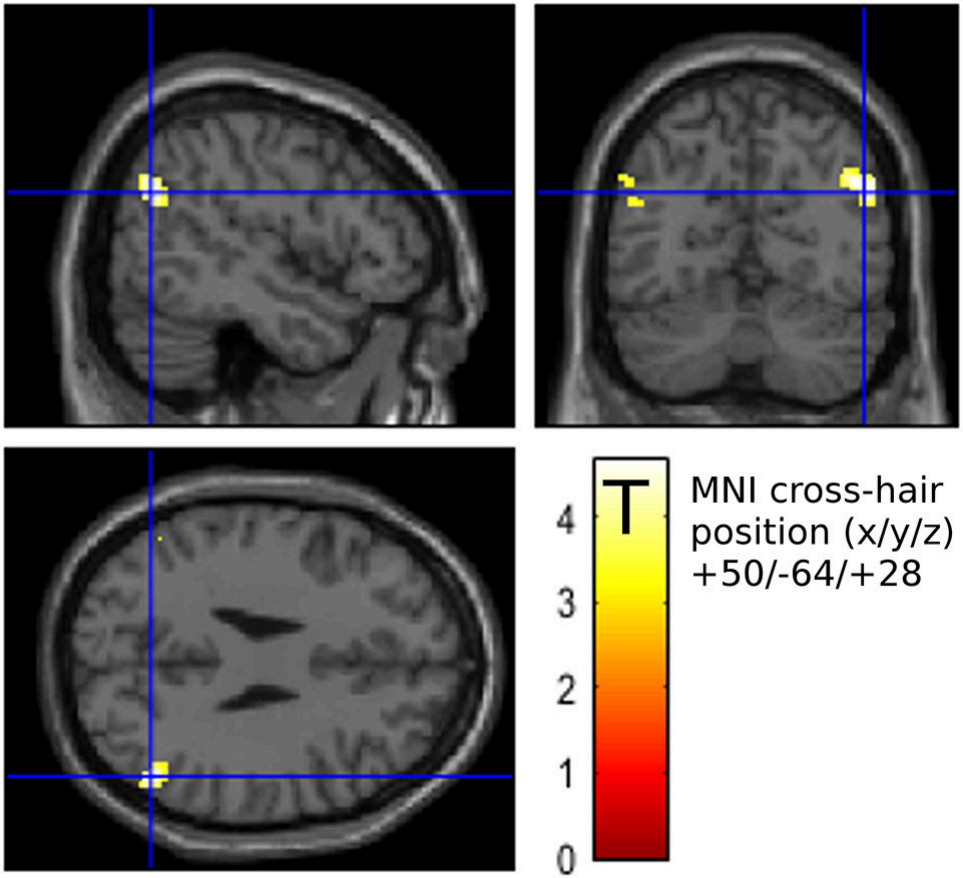
Activation difference (after – before HNMT) yielded stronger effects in chronic (*n* = 21) compared to recent-onset (*n* = 21) patients in the mask covering the left and the right angular gyrus (AG) (*p* < 0.001 uncorrected; 20 voxels extent threshold). The maximum peak was situated in the right AG (crosshairs, neurological view). This cluster peaked in a T score of 4.7 that was significant after FWE correction on cluster level (*p* < 0.05).

Based on the fact of AG involvement in conscious attention control, a region of interest (ROI) including the left and the right AG was derived from the brain atlas for Automatic Anatomical Labeling (AAL, Tzourio-Mazoyer et al., 2002) using the WFU PickAtlas (Maldjian et al., 2003). The neural base of the observed behavioral differences between long-term tinnitus sufferers and recent-onset tinnitus patients was investigated within this ROI by utilization of the HNMT effects on the distress symptoms. A flexible factorial model for repeated measures with “Time” as within-subject factor and “Group” as between-subject factor was performed in treated patients to reveal effects from the individual tinnitus duration on change in AG activation. Contrasts of the respective activation levels at T1 and T2 exhibited higher activation differences in the right AG (MNI: +50/−64/+28) and left AG (MNI: −50/−68/+24) in chronic patients compared to recent-onset patients (Figure 5). The maximum peak regarding this activation difference was located in the right AG, even for a whole-brain analysis (Table 2). When reversely contrasting the differences in the opposite direction, there were no clusters peaking higher in recent-onset than in chronic patients.

## Discussion

The effect of auditory discrimination training and sound-based stimulation have often been discussed in tinnitus therapy (Flor et al., 2004; Herraiz et al., 2010; Hoare et al., 2013) yielding inhomogeneous outcome. While most music-based approaches are passive interventions which are based on listening to computer-modified musical stimuli, the HNMT pursues an active engagement by means of repeated stimulation of the central auditory pathway with sounds in the harmonic spectrum of the individual tinnitus frequency. Thus, the therapeutic sounds are actively performed in aware attention, not as passive listening. Evidence seems to indicate that the amount of attention dedicated to the auditory input is essential (Argstatter et al., 2015). The HNMT combines complementary receptive and active based music interventions to intervene at different levels of the neural tinnitus network. The “Neuroauditive Cortex Training” is a targeted and active auditory training focusing on tone sequences centered around the frequency of the patients' tinnitus frequency. A systematic and targeted training of inaccurately intonated musical sounds enables the patients to exert influence on their auditory processes since they learn to actively filter out irrelevant information and to concentrate on relevant acoustic stimuli. Music-based exercises for mindfulness then promotes desensitization of tinnitus perception. The individual tinnitus sound is embedded in relaxation music and the patients learn to decouple possible negative reactions toward the tinnitus sound or even to completely ignore the acoustic on-top-interference. Being able to disengage from emotional stimuli may reduce the tendency to experience negative affect and redeploying attention has been postulated to lead to a “flexibility of attention” which may free up cognitive resources (Linehan et al., 2007).

Jonker et al. (2013) were able to show that auditory distractors did not provoke a general influence on the visual attention as measured by CPT. This may mean that neither the tinnitus percept nor the MRI noise themselves would be sufficient to induce attention-related effects on the visual perception. However, our results from the visual GO/NOGO attention task yielded rising frequency of omission errors (GO errors) with the duration of tinnitus. In addition, erroneous NOGO responses were reduced with the duration of tinnitus. This observation can be explained in the context of a tinnitus-duration-dependent attention loss relating to the visual task (Husain et al., 2015) due to the shift toward the auditory tinnitus percept (Alpini and Cesarani, 2006). One can then assume that difficulties in monitoring the visual stream concerned both GO and NOGO trials. If the “alarm stimulus” was perceived to a lesser degree in chronic tinnitus patients, the reduced number of false alarms can be interpreted as due to a lack of visual attention rather than from better inhibition control. Following this line of argument, the conjunction of both conditions as a common predictor for visual attention seemed justifiable for the functional analyze regarding the visual cue “O.”

**Figure 6:**
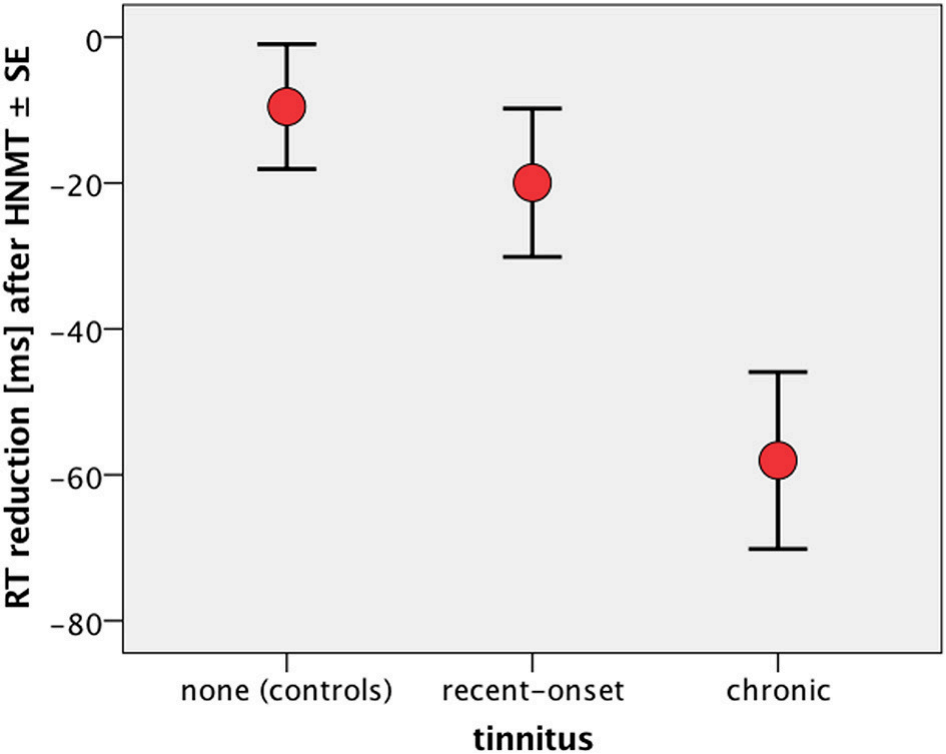
RT difference (after – before) over the 1-week study period in the GO condition. “Treated” healthy participants served as active controls (*n* = 22) to compare the mental acceleration due to the compact HNMT in treated patients with recent-onset (*n* = 21) and chronic tinnitus (*n* = 21). RT was significantly reduced in chronic patients compared to both recent-onset patients (Mann–Whitney-*U* tests; *p* < 0.05) and active controls (*p* < 0.005). There was no significant RT difference between active controls and recent-onset patients (*p* > 0.63).

Since the attention-related effects as observed by the GO/NOGO task were different with the tinnitus duration, we compared the visual attention and its change due to HNMT in recent-onset and chronic tinnitus patients. HNMT led to a decrease of omission errors in the chronic patients whose attention was most impaired before therapy. The error reduction can be explained in terms of a reinforcement of visual attention. According to this reduction of GO errors response times for the GO condition were also most reduced in chronic tinnitus patients after HNMT (Figure 6). The therapy-induced acceleration in cases of chronic tinnitus differed significantly from “treated” controls (Mann–Whitney-*U* tests; Z = −2.99; *p* < 0.005) as well as from treated patients with recent-onset tinnitus (*Z* = −2.25; *p* < 0.05). Hence, the therapy-induced alteration of attention parameters was dependent on the tinnitus duration. In contrast to this, the therapy-induced TQ change showed no influence from tinnitus duration (Figure 1).

The behavioral effect of HNMT coincidentally matched with a higher task-related activation level of the bilateral AG with a right-sided maximum. The role of the AG has previously been established in visual attention control (Taylor et al., 2011; Humphreys and Chechlacz, 2015) and it has been recognized as part of the “tinnitus core” (De Ridder et al., 2014). Dehaene and Changeux (2011) assumed its role as part of a “Global Neural Workspace” for conscious processing. Thus, one can assume that the structural overlap between visual attention network, awareness network, and tinnitus core network mediated between the duration of tinnitus and a diminished awareness in visual attention (Roberts et al., 2013). The evidence of a HNMT-induced reinforcement of AG activation in chronic tinnitus patients underlined the role of this brain area in the context of the central attention control. Therefore, the reduction of omission errors can be explained as a relaxation of the erroneous attention binding to the auditory sensation in chronic tinnitus patients.

In general, the AG is considered to be a cerebral cross-modal hub for multisensory information and for reorienting attention to relevant information (Seghier, 2013). This means that the AG acts as a central gate for reorienting or shifting the attention toward those sensations that gain the highest salience in terms of emotional value or individual meaning (Gottlieb, 2007). The AG seems to include at least two centers: a dorsal and a ventral part (Uddin et al., 2010) which are involved either in multisensory bottom-up tasks, such as reading (Price, 2010; Segal and Petrides, 2013), or in top-down control of self-referential mental concepts (Bahnemann et al., 2010; Kim, 2010). The reactivation of AG found in this study can be anatomically assigned to the ventral part of AG. This region additionally overlaps with parietal areas of the “default-mode network” (Raichle, 2015) and it plays a role in self-relevant memory retrieval (Kim, 2010) as well as in emotional and social perception (Bahnemann et al., 2010; Igelström et al., 2016). Plewnia et al. (2007) located the tinnitus-related activation of right AG (MNI: 52/−66/32) neighboring our findings of task-related reinforcement of the AG activity (MNI: 50/−64/28). This means that the same region is addressed by both monitoring the tinnitus and visual attention control, however, the attention is driven toward different sensory modalities. When suffering from chronic tinnitus, paying attention to a visually presented alarm stimuli seems to be impaired due to a failure to switch the attention toward the visual input. Trevis et al. (2016) recently conformed this finding also using a letter-based behavioral experiment. The long-term effects from the tinnitus duration causing the difficulties in attention switching may be based on structural change among attention-related and self-referencing brain networks (Lanting et al., 2016; Leaver et al., 2016). HNMT has been found to partially redirect these structural brain alterations (Krick et al., 2015). This in turn may explain the sensitivity of HNMT regarding the restored ability to switching attention between auditory and visual perception in chronic tinnitus sufferers.

Since we used temporal jittered ITI, the occurrence of the rare alarm stimuli (60 cues/1,800 letters = 3.33%) was not predictable by the participants. Following the dual-attention model (Corbetta and Shulman, 2002), the participants had to monitor the visual stream by internally guided attention (“top-down control”), whereas the occurrence of an “O” did not match the participant's expectance but happened externally driven (“bottom-up process;” Serences et al., 2005). Hence, regarding more the alarm stimuli rather than the GO/NOGO conditions, this task was similar to an oddball paradigm (Clark et al., 2000). However, activation of the inferior parietal cortex in an oddball task presupposes a relevance to the main task (Downar et al., 2001; Cabeza et al., 2012) that had been defined in turn by the required GO/NOGO decisions. In most studies on perceptual reorienting tasks the naming of the respective inferior parietal areas was traditionally named “tempero-parietal junction” (TPJ) applying a less exact anatomical assignment by subsuming parts of AG, marginal gyrus (MG), and superior temporal areas (STG; Cabeza et al., 2012). In contrast, language-related studies tend to call the same area “angular gyrus” (Cabeza et al., 2012). In this study, the assignment of relevant meaning to the oddball stimulus “O” was realized by letters that in turn may explain the focus on AG but not MG or STG when performing a whole brain analysis (Table 2).

The combination of abnormal attention control due to mental focus on an unpleasing percept has been also observed in adults with ADHD symptoms that augmented the predisposition for chronic pain, especially in coincidence with further mental disorders (Stickley et al., 2016). The association between suffering from aversive sensations and attention difficulties may play a fundamental role in chronic tinnitus sufferers as well (Husain et al., 2015). Hence, the interaction between conscious tinnitus experience and deviant attention control may also become relevant in both future tinnitus research and tinnitus treatment.

### Limitations

The compared samples of participants, suffering from either chronic or recent-onset tinnitus, differed slightly in age which was 4.5 years higher on average in chronic patients compared to recent-onset participants. However, the age of chronic patients was not correlated with the respective tinnitus duration. The age difference is due to the circumstance that chronic patients suffered from tinnitus over a comparable mean time span. When acting on the assumption of a comparable mean age of onset in both groups, the cohorts seemed representative.

## Conclusion

Our observations and the hitherto existing knowledge about the AG lead to the conclusion that this region plays a crucial role in both chronic tinnitus and suitable therapies. In particular, chronic tinnitus impaired the patients' attention for a visual task. HNMT has been shown to reinforce visual attention in chronic tinnitus patients by reorienting the activity in the AG to the cognitively demanding task. These effects regarding the role of AG in tinnitus were dependent on the time since the onset of tinnitus. The observed duration-related attention shift indicates a variable of tinnitus diversity that should be considered in therapy concepts.

## Author contributions

CK: MRI measurements, analysis of MRI data, data interpretation. HA: Development of Heidelberg Neuro-Music Therapy, therapy management, statistics. MG: therapist, analysis of clinical data, statistics. PK: tinnitus diagnostics, clinical therapy control. WR: neuroradiological screening, coordinator between the facilities, study coordinator.

### Conflict of interest statement

The authors declare that the research was conducted in the absence of any commercial or financial relationships that could be construed as a potential conflict of interest.
